# Editorial: Critical care nephrology: a multiorgan subspecialty in the ICU

**DOI:** 10.3389/fneph.2023.1269846

**Published:** 2023-08-18

**Authors:** Rolando Claure-Del Granado, Javier A. Neyra

**Affiliations:** ^1^ Division of Nephrology, Hospital Obrero No 2 – Caja Nacional de Salud (CNS), Cochabamba, Bolivia; ^2^ Instituto de Investigaciones Biomédicas e Investigación Social (IIBISMED) Facultad de Medicina, Universidad Mayor de San Simon, Cochabamba, Bolivia; ^3^ School of Medicine, University of Alabama at Birmingham, Birmingham, AL, United States

**Keywords:** critical care nephrology, acute kidney injury, renal replacement therapy (RRT), intensive care unit (ICU), nephrologist

Critical care nephrology is an emerging specialty that focuses on the management of acute kidney injury (AKI) and renal complications in critically ill patients. This field plays a pivotal role in the multidisciplinary care of patients in intensive care units (ICUs) and has gained significant recognition in recent years (Griffin et al., 2020). With the incidence of AKI on the rise and its association with increased morbidity and mortality, it is imperative to shed light on the importance of critical care nephrology in optimizing patient outcomes.

AKI is a common occurrence in critically ill patients and is often a consequence of acute stressors, such as sepsis, major surgery, or drug toxicity. Its development can have devastating consequences on patients’ overall health and can lead to multiorgan dysfunction and failure. By promptly recognizing, risk-classifying and managing AKI, critical care nephrologists can significantly impact patient outcomes.

Critical Care Nephrology has important roles in the ICU. One of the primary responsibilities of critical care nephrologists is the early detection and diagnosis of AKI. Through close monitoring of patients’ renal function, they can identify subtle changes indicative of kidney injury and intervene promptly. Emerging implementation of biomarkers of kidney stress and early injury, as well as big data-driven dynamic clinical decision support systems, can assist with this role in the ICU. Timely recognition of AKI allows for the implementation of appropriate management strategies to prevent further damage and improve clinical and patient-centered outcomes (Birkelo, 2022). The provision of Renal Replacement Therapy (RRT), including hemodialysis, peritoneal dialysis, and continuous renal replacement therapy, is a cornerstone of critical care nephrology. RRT helps to maintain fluid and electrolyte balance, remove metabolic waste products, and regulate acid-base status in patients with severe AKI and/or other organ failure (Neyra and Goldstein, 2018). Critical care nephrologists play a vital role in determining the appropriate timing, modality, dose, and duration of RRT based on individual patient characteristics and clinical phenotypes, ultimately striving to restore renal function whenever possible (Ruiz et al., 2020). Among the ICU care ecosystem, critical care nephrologists are essential members of the multidisciplinary ICU team. They collaborate closely with intensivists, surgeons, pharmacists, nurses, and other health care providers to deliver comprehensive care for critically ill patients. By actively participating in rounds and discussions, critical care nephrologists offer valuable insights into renal management, medication adjustments, fluid balance optimization, and extracorporeal multiorgan support, ensuring a holistic approach to patient care. Beyond the direct management of AKI in the ICU, critical care nephrologists actively engage in research and initiatives aimed at preventing renal complications and advancing blood purification technologies in critically ill patients. They contribute to the development of evidence-based protocols and guidelines for renal protection, advocate for improved patient safety measures, and investigate novel therapies to mitigate the impact of AKI on patient outcomes. Through their efforts, critical care nephrologists continually push the boundaries of knowledge and promote advancements in the field.

The development and testing of extracorporeal blood purification therapies also constitute a novel area in which critical care nephrologists are deeply involved (Monard, 2019). The use of various devices for immunomodulation, mitigation of inflammation by removal of endotoxins or cytokines, and direct pathogen removal from blood have regained recognition during the COVID-19 pandemic. While multiple studies are underway in patients with sepsis and other critically ill populations, the provision of these therapies to the right patient and the right time represents an area of future promise.

While critical care nephrology has made significant strides, several challenges persist. Limited resources, shortage of trained nephrologists, and financial constraints often pose barriers to optimal care delivery. To overcome these challenges, increased awareness and investment in critical care nephrology education and training programs are essential. Governments, healthcare organizations, and academic institutions must collaborate to address these gaps and ensure that critically ill patients receive the highest standard of nephrology care while in the ICU. The recent COVID-19 pandemic underpinned the crucial role of critical care nephrologists in the planning of demand and capacity projections for the optimal provision of RRT in the ICU (Neyra, 2020). Therefore, critical care nephrologists should be integral members of quality assurance systems in the ICU.

Furthermore, harnessing the potential of technology, digital health, and telemedicine can revolutionize critical care nephrology. Remote monitoring, teleconsultations, and artificial intelligence-guided decision support systems can enable nephrologists to provide expert advice to ICU teams in resource-limited settings. Such technological advancements have the potential to bridge the gap in access to specialized care, especially in underserved regions (Selby, 2022).

In summary, critical care nephrology plays a vital role in optimizing patient outcomes in the ICU setting. Through early detection and diagnosis of AKI, timely patient selection and initiation of RRT, RRT-guided fluid de-resuscitation, and active participation in multidisciplinary care, critical care nephrologists contribute to the overall well-being of critically ill patients at different stages of ICU care. As the field continues to evolve, it is crucial to address challenges, promote education, collaboration and research, and embrace technological advancements to ensure that all patients, regardless of their location or available resources, receive the highest value-based and quality critical care nephrology services. By doing so, we can bridge the gap and make significant strides towards improving patient outcomes and care processes in critical care nephrology.

## Author contributions

RC-G: Writing – original draft, Writing – review & editing. JN: Writing – review & editing.
